# Advanced metabolic engineering strategies for increasing artemisinin yield in *Artemisia annua* L.

**DOI:** 10.1093/hr/uhad292

**Published:** 2024-01-02

**Authors:** Yongpeng Li, Yinkai Yang, Ling Li, Kexuan Tang, Xiaolong Hao, Guoyin Kai

**Affiliations:** Zhejiang Provincial TCM Key Laboratory of Chinese Medicine Resource Innovation and Transformation, Zhejiang International Science and Technology Cooperation Base for Active Ingredients of Medicinal and Edible Plants and Health, Jinhua Academy, School of Pharmaceutical Sciences, Academy of Chinese Medical Sciences, Zhejiang Chinese Medical University, Hangzhou 310053, China; Zhejiang Provincial TCM Key Laboratory of Chinese Medicine Resource Innovation and Transformation, Zhejiang International Science and Technology Cooperation Base for Active Ingredients of Medicinal and Edible Plants and Health, Jinhua Academy, School of Pharmaceutical Sciences, Academy of Chinese Medical Sciences, Zhejiang Chinese Medical University, Hangzhou 310053, China; Frontiers Science Center for Transformative Molecules, Joint International Research Laboratory of Metabolic and Developmental Sciences, Plant Biotechnology Research Center, Fudan-SJTU-Nottingham Plant Biotechnology R&D Center, School of Agriculture and Biology, Shanghai Jiao Tong University, Shanghai 200240, China; Frontiers Science Center for Transformative Molecules, Joint International Research Laboratory of Metabolic and Developmental Sciences, Plant Biotechnology Research Center, Fudan-SJTU-Nottingham Plant Biotechnology R&D Center, School of Agriculture and Biology, Shanghai Jiao Tong University, Shanghai 200240, China; Zhejiang Provincial TCM Key Laboratory of Chinese Medicine Resource Innovation and Transformation, Zhejiang International Science and Technology Cooperation Base for Active Ingredients of Medicinal and Edible Plants and Health, Jinhua Academy, School of Pharmaceutical Sciences, Academy of Chinese Medical Sciences, Zhejiang Chinese Medical University, Hangzhou 310053, China; Zhejiang Provincial TCM Key Laboratory of Chinese Medicine Resource Innovation and Transformation, Zhejiang International Science and Technology Cooperation Base for Active Ingredients of Medicinal and Edible Plants and Health, Jinhua Academy, School of Pharmaceutical Sciences, Academy of Chinese Medical Sciences, Zhejiang Chinese Medical University, Hangzhou 310053, China

## Abstract

Artemisinin, also known as ‘Qinghaosu’, is a chemically sesquiterpene lactone containing an endoperoxide bridge. Due to the high activity to kill *Plasmodium parasites*, artemisinin and its derivatives have continuously served as the foundation for antimalarial therapies. Natural artemisinin is unique to the traditional Chinese medicinal plant *Artemisia annua* L., and its content in this plant is low. This has motivated the synthesis of this bioactive compound using yeast, tobacco, and *Physcomitrium patens* systems. However, the artemisinin production in these heterologous hosts is low and cannot fulfil its increasing clinical demand. Therefore, *A. annua* plants remain the major source of this bioactive component. Recently, the transcriptional regulatory networks related to artemisinin biosynthesis and glandular trichome formation have been extensively studied in *A. annua*. Various strategies including (i) enhancing the metabolic flux in artemisinin biosynthetic pathway; (ii) blocking competition branch pathways; (iii) using transcription factors (TFs); (iv) increasing peltate glandular secretory trichome (GST) density; (v) applying exogenous factors; and (vi) phytohormones have been used to improve artemisinin yields. Here we summarize recent scientific advances and achievements in artemisinin metabolic engineering, and discuss prospects in the development of high-artemisinin yielding *A. annua* varieties. This review provides new insights into revealing the transcriptional regulatory networks of other high-value plant-derived natural compounds (e.g., taxol, vinblastine, and camptothecin), as well as glandular trichome formation. It is also helpful for the researchers who intend to promote natural compounds production in other plants species.

## Introduction

Malaria is a devastating mosquito-borne disease caused by five *Plasmodium* species: *Plasmodium falciparum*, *Plasmodium vivax*, *Plasmodium malariae*, *Plasmodium ovale* and *Plasmodium knowlesi*, among which *P. falciparum* and *P. vivax* are the most threatening species [1]. As reported by WHO [2], an estimated 247 million new cases of malaria were observed worldwide, leading to 619 000 deaths. It is noteworthy that the African region took up about 95% and 96% of global malaria cases and deaths, respectively, while *P. falciparum* is the most lethal and most prevalent malaria parasite. Generally, malaria transmission occurs through female *Anopheles* mosquito bites or blood transfusion from a malaria-infected donor [3]. People with malaria often develop or present flu-like symptoms of fever, chills, and headache during the early stage of infection, and are thereby difficult to recognize and diagnose [4]. If left untreated, they may experience severe complications and die [5]. Many efforts have been expended to reduce the global burden of malaria, with valid vector control and the usage of preventive antimalarial therapies showing the most profound impact [6].

Artemisinin-based combination therapies (ACTs) containing one artemisinin derivative component (for example, artemether, artesunate or dihydroartemisinin) and other antimalarial drugs (for example, mefloquine, lumefantrine, amodiaquine, piperaquine, and pyronaridine) have been proposed as the best available treatment, particularly for *P. falciparum* malaria [7, 8]. The main active ingredient of ACTs, artemisinin is specifically produced by a traditional Chinese medicine Qinghao, whose original plant *Artemisia annua* is an Asteraceae plant [9]. As an ancient herb, the use of Qinghao to fight malaria can be dated back to 2000 years ago [10]. In several traditional Chinese medical literatures such as *Zhou Hou Bei Ji Fang*, *Ben Cao Gang Mu* and *Wenbing Tiaobian*, Qinghao has been documented to relieve malaria symptoms such as periodic fevers [7]. However, the principle of its antimalarial property was unknown until the Chinese scientist Youyou Tu and her colleagues determined the single active ingredient artemisinin in crystal form in 1972 [11]. Due to the distinguished contribution in the discovery of artemisinin, which has saved countless lives, professor Youyou Tu shared the Nobel Prize in Physiology or Medicine in 2015 [12].

**Figure 1:**
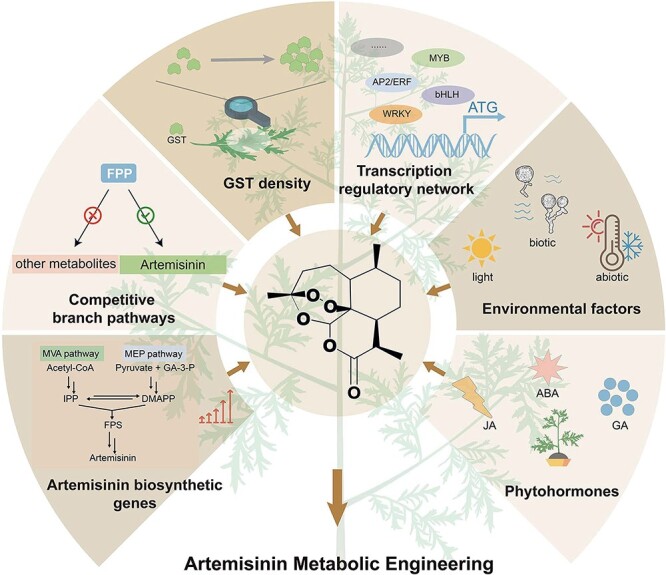
Metabolic engineering strategies for artemisinin biosynthesis in *Artemisia annua*

Artemisinin is a chemically sesquiterpene lactone with an endoperoxide bridge which greatly contributes to its antimalarial action. It was found that the cleavage of the endoperoxide bond in the structure of artemisinin leads to the formation of highly reactive carbon-centered radicals, thereby eradicating the *Plasmodium* parasites [13, 14]. In addition, accumulated evidences demonstrated that artemisinin and its derivatives showed good therapeutic effect for the treatment of cancer, diabetes, fibrosis, inflammation, viral infection, as well as autoimmune disease [15–19]. Nevertheless, the artemisinin production of wild type *A. annua* plants is low, accounting for 0.1–1% (dry weight, DW), which significantly limits its commercialization as a drug [20]. To meet the increasing demand for artemisinin, many efforts have been attempted on its synthesis using heterologous systems such as yeast, tobacco, and *Physcomitrium patens*. However, the artemisinin yield using heterologous hosts is low and has limited capacity to support the global clinical demand. Therefore, the major resource of artemisinin production remains the filed *A. annua* plants [21]. This review summarizes the metabolic engineering strategies used for the improvement of artemisinin yields in *A. annua* including enhancing the metabolic flux in the artemisinin biosynthetic pathway, blocking competition branch pathways, increasing peltate glandular secretory trichome (GST) density, using transcription factors (TFs), applying exogenous factors, and plant hormones (Fig. 1). In addition, perspectives on the future artemisinin metabolic engineering in *A. annua* are briefly discussed.

## Enhancing the metabolic flux in the artemisinin biosynthetic pathway

### Artemisinin biosynthetic pathway

**Figure 2:**
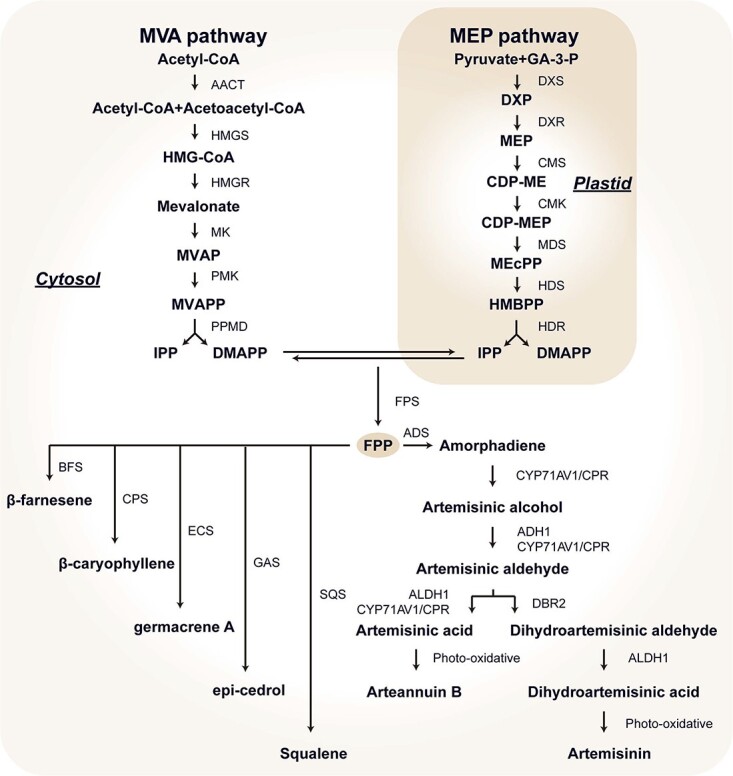
Artemisinin biosynthetic pathway in *Artemisia annua.* ADH1, alcohol dehydrogenase 1; ADS, amorpha-4,11-diene synthase; ALDH1, aldehyde dehydrogenase 1; BFS, β-farnesene synthase; CPR, cytochrome P450 oxidoreductase; CPS, β-caryophyllene synthase; CYP71AV1, cytochrome P450 monooxygenase; DBR2, artemisinic aldehyde Δ11(13)-reductase (double bond reductase 2); FPS, farnesyl pyrophosphate synthase; GAS, germacrene A; SQS, squalene synthase.

Artemisinin is produced exclusively in the peltate GSTs distributed on its aerial parts such as leaf, stem, and inflorescence of *A. annua* plants. Recent efforts by several research groups have established a basic understanding of the artemisinin biosynthesis pathway. While being a sesquiterpene, artemisinin is synthesized through the isoprenoid biosynthetic pathway, in which farnesyl pyrophosphate (FPP) acts as a common intermediate precursor (Fig. 2). FPP is formed by involving sequential head-to-tail condensation of two molecules of isopentenyl diphosphate (IPP) with one molecule of its isomer dimethylallyl diphosphate (DMAPP), and this reaction is catalyzed by farnesyl pyrophosphate synthase (FPS) [22]. In plants, IPP and DMAPP, the basic five-carbon building block units of isoprenoids are synthesized from two distinct pathways: the cytosolic mevalonate (MVA) pathway and the plastid-localized methylerythritol phosphate (MEP) pathway [23]. The biosynthesis of artemisinin begins with the lyase activity of amorpha-4,11-diene synthase (ADS), which cyclizes the substrate FPP to synthesize amorpha-4,11-diene as the first committed and rate-limiting step [24]. Amorpha-4,11-diene is hydroxylated to artemisinic alcohol, then oxidised to artemisinic aldehyde and artemisinic acid (AA). These three reactions are processed by the multi-function cytochrome P450 monooxygenase (CYP71AV1) and its cognate reductase cytochrome P450 oxidoreductase (CPR) [24–28]. It is suggested that the conversion of AA to arteannuin B is a nonenzymatic photo-oxidative reaction [29]. On the other hand, artemisinic aldehyde Δ11(13)-reductase, also called double bond reductase 2 (DBR2) catalyzes the synthesis of dihydroartemisinic aldehyde from artemisinic aldehyde. Based on the oxidation activity of aldehyde dehydrogenase 1 (ALDH1), dihydroartemisinic aldehyde is transformed into dihydroartemisinic acid (DHAA), which is the direct precursor of artemisinin. Similar to the production of arteannuin B, artemisinin was generated from DHAA by a series of spontaneous autoxidation reactions in an enzyme-independent manner [30, 31]. In addition, it has recently been reported that alcohol dehydrogenase 1 (ADH1) and ALDH1 are also associated with the production of artemisinic aldehyde and AA, respectively [32, 33].

### Artemisinin biosynthetic pathway genes

Promoting the transcript levels of structural artemisinin biosynthetic pathway genes in *A. annua* plants represents an efficient method to enhance artemisinin production. AaADS, which is exclusively expressed in *A. annua* peltate GSTs, catalyzes the beginning step of the artemisinin biosynthetic pathway [34, 35]. In *AaADS*-silenced *A. annua* lines, the artemisinin yield was reduced by 95% as compared with the WT plants, indicating the crucial role of AaADS in biosynthesizing artemisinin [36]. As expected, overexpression of *AaADS* results in a marked enhancement of artemisinin yield [37]. Later, *A. annua* plants overexpressing *AaCYP71AV1* and its redox partner *AaCPR* were generated, which show 38% higher artemisinin yield than the control plants [38]. Interestingly, overexpression of *AaDBR2* in *A. annua* plants not only remarkably enhanced the contents of artemisinin and DHAA, but also led to higher accumulation of AA and arteannuin B, which is located in the competition branch pathway of artemisinin biosynthesis [39].

### Upstream pathways genes

MVA and MEP pathways provide the initial building blocks for the formation of isoprenoid in higher plants. In *A. annua* plants, artemisinin biosynthesis mainly utilizes carbon blocks from the MVA pathway, in which HMG-CoA reductase (HMGR) catalyzes the rate-limiting step [40]. Overexpression of the *Catharanthus roseus HMGR* resulted in increased artemisinin production by 22.5–38.9% as compared with the non-transgenic control [41, 42]. Enhancing the metabolic of the MEP pathway has a similar effect. For instance, the *AaHDR1-*overexpressing *A. annua* lines showed a significant increase in artemisinin, arteannuin B, and other sesquiterpenes, as well as several monoterpenes, whereas the *AaHDR1-*antisense lines led to opposite results [43]. Considering that FPS has important roles in regulating sesquiterpenoid biosynthesis, *FPS* genes from *Gossypium arboreum* and *A. annua* were transformed into *A. annua* plants, respectively. The highest artemisinin concentration in the *FPS*-overexpressing plants was about 10 mg/g (DW) [44, 45].

### Multigene engineering

In recent years, multigene engineering which allows the simultaneous transfer of several genes to host’s genome has been widely used to trigger artemisinin production. By overexpressing *C. roseus HMGR* together with *AaADS*, the artemisinin yield was elevated up to 1.73 mg/g (DW), which was 7.65-fold higher than the wild-type plants [46]. In the transgenic *A. annua*, decreased levels of squalene, the precursor of sterol biosynthesis was also observed, which might result from the enhanced accumulation of *AaADS* transcripts [47]. Co-overexpression of the *AaHMGR* and *AaFPS* remarkably enhanced the artemisinin production, with the highest artemisinin content (about 9 mg/g DW) being 1.8-fold of that of the WT *A. annua* [48]. Additionally, the transgenic *A. annua* lines overexpressing *AaFPS*/*AaDXR*, *AaCYP71AV1*, and *AaCPR* accumulated upregulated levels of artemisinin [49, 50]. By overexpressing *AaADS*, *AaCYP71AV1*, and *AaCPR* in a high-artemisinin cultivar of *A. annua*, the highest artemisinin production reached 15.1 mg/g (DW) [51]. To further enhance the artemisinin biosynthesis, Shi *et al*. generated a multigene expression construct consisting of *AaADS*, *AaCYP71AV1*, *AaCPR*, and *AaALDH1*, which was stably transformed to *A. annua* [52]. Most transgenic plants showed increased artemisinin content, with Line 63 having the highest artemisinin concentration of 27 mg/g (DW), about 3.4-fold of that of WT plants [52]. It is noteworthy that simultaneous overexpression of *AaHMGR*, *AaFPS*, and *AaDBR2* greatly improved the artemisinin yield, among which HFD82 had the most abundant artemisinin content of 32 mg/g (DW) [53]. Most recently, increased artemisinin production was obtained by reconstructing the artemisinin biosynthetic pathway and enhancing GST formation, with the most abundant artemisinin production being 24.7 mg/g (DW) [54]. These findings suggested that enhancing the artemisinin biosynthetic pathway metabolic flux, especially involving multigene engineering ,was a promising strategy for artemisinin synthesis.

## Blocking the competition branch pathways

FPP is a metabolic intermediate for the biosynthesis of terpenoids, terpenes, and steroids. In plants, FPP can be converted into various sesquiterpenes such as β-caryophyllene, β-farnesene, and germacrene A by their sesquiterpene synthases including β-caryophyllene synthase (CPS), β-farnesene synthase (BFS), and germacrene A synthase (GAS). Additionally, squalene synthase (SQS) catalyzes the cyclization of two molecules of FPP to yield squalene, a polyunsaturated hydrocarbon of the triterpene type, which is the precursor of steroids. Blocking these competition branch pathways against the artemisinin biosynthetic pathway helped to direct the metabolic flux into artemisinin biosynthesis, thereby leading to improved artemisinin yields. Using hairpin-RNA-mediated gene silencing, the *SQS* transcript levels were firstly downregulated [55, 56]. Among the generated transgenic lines, s159C had the highest level of artemisinin yield (31.4 mg/g), approximately 2.14-fold higher than the WT *A. annua* [55]. Additionally, suppression of *CPS* led to increased artemisinin production [57]. Encouraged by these results, four terpenoids including β-caryophyllene, β-farnesene, germacrene A, as well as squalene synthetic pathways were respectively blocked by suppressing the expression of their corresponding encoding genes *CPS*, *BFS*, *GAS*, and *SQS* using the antisense method [58]. The artemisinin yield in the obtained transgenic lines was elevated by 71–103%.

## Transcriptional regulatory networks

TFs are proteins that could bind to the *cis*-regulatory sequences in the promoter of target genes, thereby activating or repressing the rate of gene transcription [59]. Targeting TFs that have the ability to simultaneously modulate multiple biosynthetic genes is of great potential for enhancing the biosynthesis of natural products [60]. Many TFs from several different TF families have been determined to be related to the artemisinin accumulation through modulating artemisinin biosynthetic pathway genes or regulating trichome initiation and development, or both. In addition to their important roles in promoting artemisinin yields, some TFs are helpful for flavonoid biosynthesis, disease resistance as well as abiotic stress tolerance in *A. annua* (Table 1).

**Table 1:** A list of TFs that have been functionally identified in *Artemisia annua*.

**TF family**	**TF name**	**Signaling pathway**	**Function**	**References**
AP2/ERF	AaERF1	JA, ET wounding	Positively regulates artemisinin biosynthesis and disease resistance	[61, 62]
	AaERF2	JA	Positively regulates artemisinin biosynthesis	[61]
	AaORA	JA	Positively regulates artemisinin biosynthesis and disease resistance	[63]
	AaTAR1	/	Positively regulates artemisinin biosynthesis and GST formation	[64]
	AaWIN1	/	Positively regulates GST formation	[65]
WRKY	AaWRKY1	JA	Positively regulates artemisinin biosynthesis	[66, 67]
	AaGSW1	JA	Positively regulates artemisinin biosynthesis	[68]
	AaGSW2	JA	Positively regulates GST formation	[69]
	AaWRKY17	JA, SA	Positively regulates artemisinin biosynthesis and disease resistance	[70]
	AaWRKY9	JA, Light	Positively regulates artemisinin biosynthesis	[71]
	AaWRKY4	/	Positively regulates artemisinin biosynthesis	[72]
bHLH	AabHLH1	JA	Positively regulates artemisinin biosynthesis	[73, 74]
	AaPIF3	/	Positively regulates artemisinin biosynthesis	[75]
	AaMYC2	JA	Positively regulates artemisinin biosynthesis	[76]
	AaMYC2-like	JA	Positively regulates artemisinin biosynthesis	[77]
	AabHLH112	JA, Cold	Positively regulates artemisinin biosynthesis	[78]
	AabHLH2	/	Negatively regulates artemisinin biosynthesis	[79]
	AabHLH3	/	Negatively regulates artemisinin biosynthesis	[79]
	AabHLH113	JA, ABA	Positively regulates artemisinin biosynthesis	[80]
bZIP	AaABF3	ABA	Positively regulates artemisinin biosynthesis	[81]
	AabZIP1	ABA	Positively regulates artemisinin biosynthesis	[82]
	AabZIP9	/	Positively regulates artemisinin biosynthesis	[83]
	AaHY5	Light	Positively regulates artemisinin biosynthesis	[84]
	AaTGA6	SA	Positively regulates artemisinin biosynthesis	[85]
	AaABI5	Light, ABA	Positively regulates artemisinin biosynthesis	[86]
MYB	AaMYB1	/	Positively regulates artemisinin biosynthesis and GST formation	[87]
	AaMIXTA1	/	Positively regulates GST formation	[52]
	AaTAR2	/	Positively regulates GST formation	[88]
	AaMYB17	/	Positively regulates GST formation	[89]
	AaMYB15	JA, Dark	Negatively regulates artemisinin biosynthesis	[90]
	AaMYB5	JA	Negatively regulates GST formation	[91]
	AaMYB16	/	Positively regulates GST formation	[91]
	AaTLR1	/	Negatively regulates GST formation	[92]
	AaTLR2	/	Negatively regulates GST formation	[92]
	AaMYB108	JA, Light	Positively regulates artemisinin biosynthesis	[93]
HD-ZIP	AaHD8	/	Positively regulates GST formation	[94]
	AaHD1	JA	Positively regulates GST formation	[95]
MADS-box	AaSEP1	JA, Light	Positively regulates GST formation	[96]
	AaSEP4	/	Positively regulates artemisinin biosynthesis	[97]
TCP	AaTCP14	JA	Positively regulates artemisinin biosynthesis	[98]
	AaTCP15	JA, ABA	Positively regulates artemisinin biosynthesis	[99]
SPL	AaSPL2	JA	Positively regulates artemisinin biosynthesis	[100]
	AaSPL9	/	Positively regulates GST formation	[101]
EIN3/EIL	AaEIN3	ET	Positively regulates artemisinin biosynthesis	[102]
YABBY	AaYABBY5	JA	Positively regulates artemisinin biosynthesis and flavonoid biosynthesis	[103, 104]
NAC	AaNAC1	JA, SA, Dehydration, Cold	Positively regulates artemisinin biosynthesis, drought tolerance and disease resistance	[105]
C2H2	AaZFP1	/	Positively regulates artemisinin biosynthesis	[106]

### AP2/ERF TF family

The APETALA2/ethylene response factor (AP2/ERF) superfamily is determined by the containment of the DNA-binding AP2/ERF domain, which is composed of about 60 to 70 amino acid residues [107]. The AP2/ERF family members play a key role in regulating plant development, stress responses as well as secondary metabolite biosynthesis [61, 108]. AaERF1 and AaERF2 from *A. annua* have proved to activate the expression of both *AaADS* and *AaCYP71AV1* by binding to the CRTDREHVCBF2 (CBF2) and RAV1AAT (RAA) *cis*-elements in their promoters, thereby promoting artemisinin production [61]. Moreover, AaERF1 is associated with defensive responsiveness and has positive roles in improving disease resistance in *A. annua* [62]. The trichome-specific AP2/ERF TF AaORA, which is exclusively expressed in the peltate and T-shaped GSTs of *A. annua*, was confirmed to be an important positive modulator of artemisinin biosynthesis, as well as disease resistance [63]. In addition to their sole function in the regulation of artemisinin biosynthetic pathway genes of those AP2/ERF members, TRICHOME AND ARTEMISININ REGULATOR 1 (TAR1), was found to function in modulating both trichome formation and the artemisinin biosynthesis [64]. Overexpression of *TAR1* in *A. annua* resulted in remarkably increased artemisinin content, whereas a sharp reduced artemisinin content was observed in *TAR1*-RNAi *A. annua* plants, in which many GSTs showed an abnormal inflated top and cell number reduction [64]. Recently, AaWIN1, an AP2/ERF member which is predominantly expressed in buds, flowers and trichomes has also been found to promote GST formation [65].

### WRKY TF family

As one of the largest TF families, WRKY TFs are reported to be involved in various biological processes in plants by forming integral parts of signaling networks [109]. WRKY proteins usually contain two highly conserved domains: a N-terminus WRKYGQK motif and a C-terminus C2H2 or C2HC zinc-finger motif [70]. A total of 122 WRKY members were genome-wide characterized in *A. annua* [110]. Many WRKYs have been shown to have function in regulating artemisinin accumulation through modulating the artemisinin biosynthetic pathway or trichome development. For example, overexpression of *AaWRKY1* led to an increase of artemisinin yield by 1.3- to 2.0-fold [66]. *In vivo* and *in vitro* assays demonstrated that AaWRKY1 could recognize and bind to the W-boxes (TTGACC) in the promoter regions of *AaADS* [66, 67]. Later, a peltate GST-specific WRKY TF AaGSW1 was functionally characterized [68]. AaGSW1 serves as a positive regulator of *AaCYP71AV1* and *AaORA* by directly activating their promoters. AaGSW1-overexpressing lines showed marked increased artemisinin and DHAA contents. Additionally, several other WRKYs including AaWRKY17, AaWRKY9, and AaWRKY4 have been also shown to positively regulate artemisinin production [70–72]. WRKY40 [110] and WRKY14 [111] were identified to have0 potential roles in modulating artemisinin biosynthesis. Recently, another peltate GST-specific WRKY TF AaGSW2 has been observed to contribute to peltate GST development, thereby enhancing artemisinin biosynthesis and accumulation [69]. Overexpression of *AaGSW2*, ectopic expression of AaGSW2-homologs from two mint cultivars in *A. annua* led to an approximately 1-fold and 0.8-fold higher peltate GST density and artemisinin content, respectively [69].

### bHLH TF family

The basic helix–loop–helix (bHLH) TF family members have two conserved motifs, a basic DNA binding region and a HLH region [112]. Several recent studies have revealed bHLH TFs had an important role in modulating artemisinin biosynthesis. Transient overexpression of *AabHLH1* and *AaMYC2-like* induced upregulated transcripts levels of many artemisinin biosynthetic genes [73, 77]. Overexpression of *AaMYC2* in *A. annua* led to elevated artemisinin accumulation. Further study revealed that AaMYC2 showed high affinity in binding to the G-box-like motifs within the promoters of *AaCYP71AV1* and *AaDBR2* [76]. Similarly, overexpression of *AaPIF3* also induced markedly improved artemisinin production [75]. AabHLH112 acts as an indirect positive regulator of artemisinin *via* binding to the *AaERF1* promoter [113]. A recent study showed AabHLH112 also played key roles in promoting the biosynthesis of other sesquiterpenes such as β-caryophyllene, epi-cedrol, and β-farnesene [78]. In addition to those positive regulators, AabHLH2 and AabHLH3, which belong to MYC-type bHLH TFs, were recently identified as negative regulators of artemisinin synthesis [79].

### bZIP TF family

The basic leucine zipper (bZIP) TF family, which is one of the largest and most conserved TF families in plants, has proved to modulate multiple biological processes [114, 115]. Zhang *et al.* (2015) demonstrated that AabZIP1 promote artemisinin production by directly activating the promoter activity of *AaADS* and *AaCYP71AV1* [82]. Another AabZIP9 has also been found to directly activate the *AaADS* promoter activity, while AaABF3 directly binds to the G-box *AaALDH1* promoter, thus promoting its expression [81, 83]. Several bZIP TFs having indirect roles in promoting artemisinin biosynthesis were also identified. For instance, in *AaHY5*-overexpresing *A. annua* the transcript levels of *AaADS*, *AaCYP71AV1*, *AaDBR2*, and *AaALDH1* were remarkably upregulated. However, AaHY5 does not bind to their promoter regions. Further results showed that *AaHY5* could directly activate the promoter of *AaGSW1*, a critical positive modulator of artemisinin production [84]. AaTGA6 belonging to the TGA class of the bZIP TF family has been proven to promote artemisinin production by directly triggering the accumulation of *AaERF1* [85]. In addition, a report has shown that AabZIP1 could directly activate the promoters of *AaMYC2*, thereby enhancing the accumulation of *AaDBR2* and *AaALDH1* transcripts [116]. Most recently, AaABI5 was reported to be involved in light and abscisic acid signaling-mediated artemisinin biosynthesis in *A. annua* [86].

### MYB TF family

The myeloblastosis (MYB) TF family is large, functionally diverse and represented in all eukaryotes. Most MYB TFs have different numbers of MYB domain repeats, which confer their ability to bind DNA. The MYB family in plants has expanded in a selective manner, especially *via* the R2R3-MYB large family [117]. In *A. annua*, many MYBs are identified to be related to trichome initiation and development. AaMYB1 is the first MYB member that was identified to have positive roles in the modulation of trichome formation [87]. Two R2R3-MYB AaMIXTA1 and AaMYB17 which is expressed predominantly in the *A. annua* GSTs could raise the GST number and artemisinin yield [89, 118]. In addition, AaTAR2 was reported to have versatile roles such as promoting trichome initiation, as well as artemisinin and flavonoid production [88]. Xie *et al.* (2021) has characterized two MYB TFs acting competitively in forming a protein complex, in which AaMYB16 promotes GST initiation, whereas AaMYB5 has the opposite effect [91]. Two R2R3 MYBs TLR1 and TLR2, which negatively regulate trichome density, were recently identified [92]. In addition to the roles in regulating GST formation, AaMYB15 was identified to be the first R2R3-MYB to restrain the production of artemisinin [90]. Most recently, AaMYB108 was reported to be a very important mediator of artemisinin production in *A. annua* by combining light and jasmonic acid (JA) signaling pathways [93].

### HD ZIP TF family

The homeodomain-leucine zipper (HD ZIP) TFs are unique to plants functioning as homo- or heterodimers [119]. Two HD-ZIP IV subfamily members, AaHD1 and AaHD8, were identified to positively regulate artemisinin production by promoting GST formation [94, 95]. AaHD8 interacts with AaMIXTA1, forming a HD-ZIP IV/MIXTA complex, which results in improved transcriptional activity in modulating the accumulation of *AaHD1* transcripts. In addition, Xie *et al.* have shown that AaHD1 acts as a positive regulator of *AaGSW2* [69]. Also, it is proved that AaMYB16/AaMYB5 and AaHD1 form a HD-ZIP-MYB protein complex that positively or negatively regulates the expression *AaGSW2*, thereby modulating trichome initiation and development [91].

### TCP TF family

TEOSINTE BRANCHED 1/CYCLOIDEA/PROLIFERATING CELL FACTOR (TCP) family members including AaTCP14 and AaTCP15 were reported to have a vital role in modulating artemisinin production [98, 99]. Both AaTCP14 and AaTCP15 interact with AaORA to form a transcriptional cascade that promotes artemisinin biosynthesis.

### MADS-box TF family

Recently, two MADS-box TF family members, AaSEP1 and AaSEP4, were functionally identified. AaSEP1 acts as an integrator of JA and light-regulated GST initiation and positively regulates GST formation, while AaSEP4 is a positive mediator of artemisinin production [96, 97].

### SPL TF family

Similar to MAD-box TFs, two SQUAMOSA Promoter-Binding Protein-Like (SPL) TF family members with distinct roles were identified in *A. annua*. AaSPL2 interacts with a ‘GTAC’ motif in the *AaDBR2* promoter, thus upregulating the expression of *AaDBR2* and leading to the improvement of artemisinin content [100]. Another SPL member AaSPL9 could bind to the ‘GTAC’ *cis*-element of *AaHD1* promoter and activate its expression, thus increasing artemisinin production by promoting glandular trichome initiation [101].

### YABBY TF family

The YABBY Family TF AaYABBY5 was reported to be associated with the biosynthesis of artemisinin by directly targeting *AaCYP71AV1* and *AaDBR2* [103]. Moreover, AaYABBY5 functions in the transcriptional modulation of flavonoid biosynthesis in *A. annua* [104].

### NAC TF family

AaNAC1 has been found to be related to stress signaling pathways in *A. annua*, as it was induced by abiotic stresses including dehydration and low temperature, as well as phytohormones such as salicylic acid (SA) and methyl jasmonate (MJ) [105]. Through genetic transformation assays, AaNAC1 has been proved to improve artemisinin production by indirectly activating the promoter of *AaADS*. In addition, overexpression of *AaNAC1* in *A. annua* and Arabidopsis contributed to improve resistance to drought and *Botrytis cinerea*.

### C2H2 TF family

Deng *et al.* identified an ABA and MeJA-induced C2H2-type TF, namely AaZFP1, as a positive modulator of artemisinin production [106]. Transient overexpression of *AaZFP1* resulted in elevated transcript levels of *AaIPPI1*, which is an enzyme-encoding gene in the upstream artemisinin biosynthetic pathways. Biochemical assays demonstrated that AaZFP1 directly activated the expression of *AaIPPI1*.

### EIN3/EIL TF family

The ethylene-insensitive3-like/ethylene-insensitive3 (EIN3/EIL) TF family members are associated with the ethylene and sulfur signaling pathways. AaEIN3 acts as a suppressor of artemisinin yield [102]. Overexpression of *AaEIN3* resulted in decreased transcript levels of *AaADS*, *AaCYP71AV1*, and *AaDBR2*, as well as *AaORA* which encodes a transcriptional activator of artemisinin biosynthetic pathway. When *AaEIN3* was suppressed, the abovementioned genes were upregulated and led to enhanced artemisinin production.

**Figure 3:**
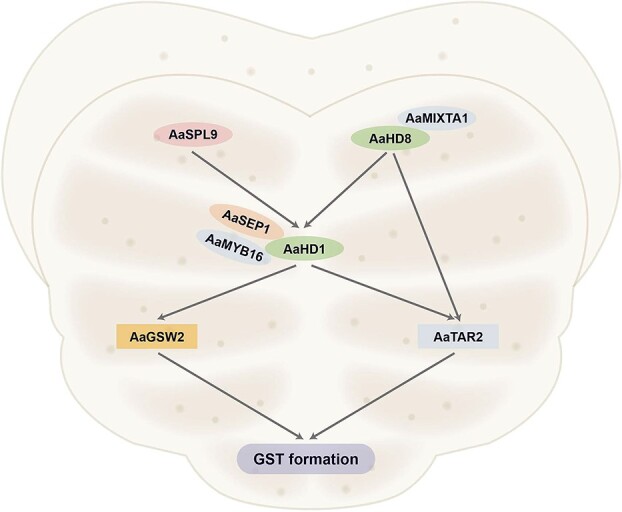
Transcriptional regulatory network of GST formation in *Artemisia annua*.

### B-box TF family

Recently, B-box (BBX) TF family in *A. annua* was genome-wide characterized. Seven *AaBBXs* were found to respond to MeJA and ABA treatment, among which AaBBX5/6/8/15/22/23 have the ability to promote the accumulation of artemisinin biosynthetic pathway genes transcripts. To characterize the function of AaBBX TF family members on modulating artemisinin biosynthesis, *AaBBX22* which exhibited highest transcript levels in the GSTs of *A. annua* was selected for the generation of transgenic *A. annua* plants. *AaBBX22-*overexpressing *A. annua* lines showed a significant increase in artemisinin content by 69% to 104% as compared with WT plants, indicating its positive role in relation to ABA and JA-modulated artemisinin production.

## GST density and its transcriptional regulatory networks

GSTs are specialized structures originated from epidermal cells on the aerial parts of many plants [120]. There are two kinds of GSTs present on the aerial parts such as leaf, stem, and inflorescence of *A. annua* plants: T-shaped and peltate GSTs. Because artemisinin has cellular toxicity to *A. annua* itself, peltate GSTs, which have subcellular spaces to sequestrate or secrete secondary metabolites, provide the ideal site for artemisinin biosynthesis [121, 122]. As the chemical factories of artemisinin, the peltate GST density in *A. annua* plants often correlates well with artemisinin production [123]. Generally, the GST formation in the genus *Artemisia* is completed at a very juvenile primordial stage of the leaf development [122]. The GST density of the mature leaves in *A. annua* is predetermined at an early primordial stage [124]. Consistently, important transcription regulators related to *A. annua* GST initiation and development are GST-specifically expressed in the very young non-expanded leaves [69].

Recently, the transcriptional regulatory networks of GST formation have been revealed (Fig. 3). AaHD1 has proved to promote GST development by directly targeting *AaGSW2* and *AaTAR2.* AaSPL9 and AaHD8 positively modulate GST formation by directly activating the expression of *AaHD1*, which encodes an important positive regulator of GST formation. Meanwhile, AaHD8 could directly promote the expression of *AaTAR2*. Moreover, several TFs function by forming a complex. For instance, AaMIXTA1 promote GST formation by interacting with AaHD8, while AaMYB16 and AaSEP1 function depending on their interaction with AaHD1.

## Environmental factors

**Figure 4:**
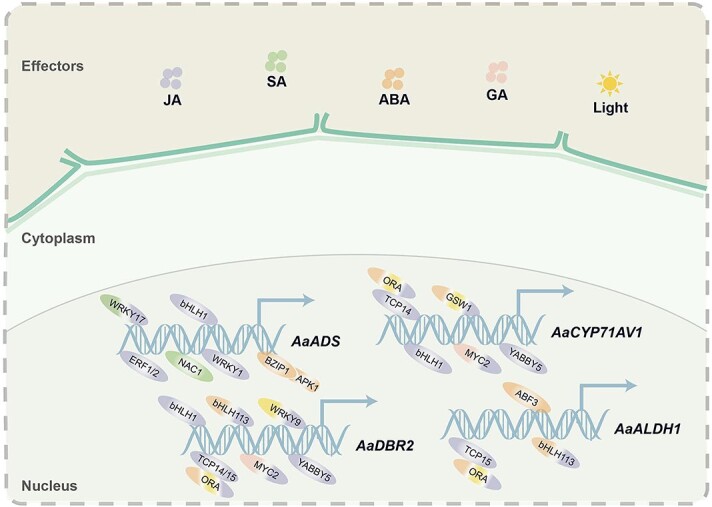
Transcriptional regulatory networks that are related to environmental factors and phytohormones-promoted artemisinin biosynthesis in *Artemisia annua*.

The biosynthesis of secondary metabolites in plants is affected by diverse environmental factors such as light, biotic, and abiotic stresses, as well as mineral nutrition and small molecule compounds. These factors often modulate artemisinin biosynthesis and/or trichome formation by activating various transcriptional regulatory networks (Fig. 4).

### Light

Light has a pivotal role in modulating plant growth, development, and secondary metabolites biosynthesis. It is demonstrated that the decrease in irradiance of sunlight led to reduced height and diameter growth, total and leaf biomass, as well as artemisinin yields [125]. The treatment of white light significantly upregulated the transcript levels of *AaADS*, *AaCYP71AV1*, *AaDBR2*, and *AaALDH1*, as well as the content of artemisinin [126]. Recent studies revealed that light could enhance artemisinin production by trigger the accumulation of several TFs transcripts such as *AaHY5*. Light spectral composition, also called light quality, also influences the biosynthesis of plant secondary metabolites [127]. Lopes et al. evaluated the effects of various light quality including blue, green, yellow and red light on artemisinin accumulation [128]. Among those tested light quality, blue light and red light function as enhancers of artemisinin production [129]. Fu et al. demonstrated that AaWRKY9, whose expression was increased dramatically by blue light and red light, has positive roles in modulating artemisinin biosynthesis [71]. Further study revealed that AaWRKY9 was directly regulated by AaHY5, while AaWRKY9 directly activated the promoter activity of *AaGSW1*. Also, AaHY5 could directly activate the expression of AaABI5 [86]. A recent study has identified another light-responsive TF AaMYB108 which positively modulates artemisinin production by forming a AaMYB108-AaGSW1 complex [93]. Moreover, promoting the expression of the blue light receptor cryptochrome1 genes such as *AtCRY1* from *Arabidopsis thaliana* and *AaCRY1* from *A. annua* effectively increases artemisinin yield [130, 131]. In addition to visible light, the ultraviolet (UV) radiation, particularly UV-B radiation, which is intrinsic to sunlight, has specific regulatory roles in plant development and acclimation responses [132, 133]. Several reports have demonstrated that low-dose, non-damaging UV-B and UV-C radiation promote artemisinin accumulation [134–137]. It has been shown that UV-B could result in a reduction of global DNA methylation level [138]. Further bisulfite sequencing PCR showed that UV-B could lead to demethylation at 4 CG-, 4 CHH-, and 2 CHG-sites of the *AaDBR2* promoter region, which might explain the upregulated expression level of *AaDBR2* under UV-B treatment [138].

### Abiotic stresses

Abiotic factors including heat, cold, salinity, drought, and waterlogging stresses have profound effects on plant growth and survival [139]. To cope with these undesirable conditions, plants have developed various physiological processes including the synthesis of primary and secondary metabolites. It is revealed that both high and low temperatures are helpful for artemisinin accumulation. Heat can promote the artemisinin biosynthesis by activating the expression of genes related to the artemisinin synthetic pathway and suppressing the expression of genes in the artemisinin-competition pathway [140]. Cold stress could trigger the accumulation of endogenous JA, thus enhancing the production of artemisinin [141]. In addition, the accumulation of artemisinin as well as essential oil was improved with prolonged exposure to salt stress [142]. It is known that water deficit significantly limits plant growth. Nevertheless, it could promote the production of secondary metabolites such as artemisinin, which relies on the plant growth stage and intensity [143, 144].

### Biotic stresses

Plant-microbe interaction is associated with plant stress resistance, and affects plant growth and secondary metabolite biosynthesis. According to the field experiment, when inoculated with two arbuscular mycorrhizal fungi (AMF) *Glomus macrocarpum* (GM) and *Glomus fasciculatum* (GF), *A. annua* plants accumulated higher contents of essential oil and artemisinin than the control plants [145]. Further data showed that the AMF inoculation can induce an increase of endogenous JA levels, thereby leading to higher transcript levels of key enzyme-encoding genes that are associated with artemisinin synthesis [146]. Moreover, the inoculation of *Piriformospora indica* is helpful for increasing the levels of artemisinin and flavonoids content in *A. annua* under arsenic stress conditions [147]. Application of endophytes has emerged as an effective alternative to chemical fertilizers for enhancing artemisinin yield [148, 149].

### Mineral nutrition and small molecule compounds

Researchers have determined the influence of mineral nutrition on biomass and artemisinin content in *A. annua*. Both organic manure and chemical fertilizers are helpful for the yield of artemisinin [150]. Appling nitrogen (N) nutrition could enhance biomass production as well as artemisinin content of *A. annua* plants; while increasing potassium (K) and phosphorus (P) application could trigger total plant biomass production but have no influence on the artemisinin yield of *A. annua* leaf [124, 151]. *A. annua* plants supplemented with boron (B) nutrition showed significantly increased leaf artemisinin concentration without influence on biomass production [151]. Application of the depolymerized form of natural bioactive agents is a novel emerging technology to promote crop growth, production as well as quality [152]. Single use, and accompanied application of irradiated sodium alginate with P and/or N fertilizers contribute to artemisinin yield [152–154]. In addition, the application of chitosan, cobalt nanoparticles, and exogenous β-ocimene could activate artemisinin biosynthesis [155–157].

## Phytohormones

In addition to those exogenous environmental factors, plant hormones such as JA, abscisic acid (ABA), gibberellin (GA), salicylic acid (SA), and strigolactone (SL) are significantly important for the modulation of plant defense, development, and secondary metabolite biosynthesis. Those plant hormones, especially JA and ABA, could trigger the transcript accumulation of a number of TFs, thus activating the artemisinin biosynthetic pathway.

### Jasmoninc acid

It is reported that *A. annua* application of exogenous methyl jasmonate (MeJA) induced the biosynthesis of several natural products such as artemisinin [158, 159]. The artemisinin content was increased by 49%, while AA and DHAA were increased by 80% and 28%, respectively, when applied with MeJA [160]. The molecular mechanisms by which JA promotes artemisinin biosynthesis have been extensively studied. JA activates the expression of *bHLH* members such as *AaMYC2*, *AaMYC2-like*, *AabHLH1*, *AabHLH112*, *AabHLH113*; *WRKY* members including *AaGSW1*, *AaWRKY9*, *AaWRKY17*; *ERF* members such as *AaORA*, *AaERF1*/*2*; *TCP* members such as *AaTCP14* and *AaTCP15*; and *SPB-Box* member *AaSPL2*. Also, JA promotes the initiation and development of *A. annua* GSTs. AaHD1 and AaGSW2 are required for JA-regulated GST formation in *A. annua*. It is demonstrated that AaHD1 could directly activate two GST initiation-related TF genes *AaGSW2* and *AaTAR2*.

### Abscisic acid

In addition, the treatment of ABA with various concentrations results in higher levels of artemisinin accumulation, as well as the key enzyme-encoding genes including *AaHMGR*, *AaFPS*, *AaCYP71AV1*, and *AaCPR* [161]. ABA activates the expression of two *AabZIP* members: *AabZIP1* and *AaABI5* [82, 86]. AabZIP1 is a direct positive modulator of *AaGSW1* and *AabH113*. These three TFs could also directly target artemisinin biosynthetic pathway genes. Moreover, AaAPK1, an ABA-responsive SnRK2-type kinase, could trigger the accumulation of artemisinin through phosphorylating AabZIP1 [162]. In addition to the positive role in artemisinin biosynthesis, exogenous ABA could mediate ROS homeostasis and maintain GST formation under copper toxicity, thereby enhancing artemisinin yield [163].

### Gibberellin

GAs has a role in the regulation of both artemisinin production and GST development in *A. annua* [164]. When treated with GA3, increased artemisinin content and decreased artemisinic acid content was observed in *A. annua*, indicating that GA3 might trigger off the transition of artemisinin acid to artemisinin [165, 166]. Gene expression analysis revealed that the transcript levels of *AaFPS*, *AaADS*, and *AaCYP71AV1* were drastically upregulated by GA3 treatment [167]. Interestingly, increased GST density was observed when GA was applied to *A. annua*. However, the molecular mechanism by which GA promote GST formation remains unclear.

### Salicylic acid

SA is the essential phytohormone related to plant defense and has the ability to promote immunity against pathogens. It is revealed that SA could induce the expression of *AaNAC1*, *AaTGA6*, and *AaWRKY17* and thus promote the yield of artemisinin [70, 85, 105, 168].

### Strigolactone

SL is widely known to have a vital role in mediating plant growth and development. In *A. annua*, foliar application of the synthetic SL analog GR24 contributes to the improvement of the GST density, thereby leading to improved artemisinin yields [169].

## Concluding remarks and future perspectives

Currently, there are still many people facing the risk of malaria infection and artemisinin serves as the first-line treatment of malaria. Given the low artemisinin content *in planta*, various strategies must be adopted to improve artemisinin production, and thereby meeting the global clinical demand. Despite considerable attempts having been made to produce artemisinin using heterologous host systems, however, *A. annua* plants remain the primary commercially feasible source of artemisinin. Here, we have overviewed the recent research progress on promoting artemisinin yield in *A. annua*. *In planta*, due to the cellular toxicity of artemisinin, its synthesis and accumulation specifically occur in the peltate GSTs which are present on the leaves, stems, and inflorescences. Therefore, triggering the accumulation of artemisinin biosynthetic pathway genes transcripts and increasing the GST density have been considered the essential ways to improving artemisinin yields in this plant.

Several studies have shown that the content of DHAA, the direct precursor of artemisinin, was sharply reduced as the leaves grow old [31, 63, 170]. Correspondingly, the transcript levels of the structural artemisinin pathway genes remarkably decreased during leaf maturation [63, 171]. According to the promoter-GUS fusion assays, the promoters of *AaADS* [35], *AaCYP71AV1* [172, 173], *AaDBR2* [174], *AaALDH1* [175], and *AaADH1* [176] were highly active in peltate GSTs of juvenile leaves, whereas no GUS staining was detected in the mature leaves. These observations indicated that in *A. annua* the biosynthesis of DHAA in old leaves was blocked, which might result from epigenetic modifications. Activating or reconstructing the artemisinin biosynthetic pathway in the peltate GSTs of old leaves represents a promising way for potentially improving artemisinin yields. Given the advantage of synthetic biology, the artemisinin biosynthetic pathways can be reconstructed in *A. annua* plants. However, limited promoters are available to drive the expression of the artemisinin biosynthetic pathway in the GSTs of mature leaves. In addition, the widely used constitutive promoter CaMV 35S has weak activity in peltate GSTs of *A. annua* [177, 178]. Recently, the tpACT promoter that shows activity in the peltate GSTs of both young and old leaves of *A. annua* has been used to promote artemisinin production [54].

Regarding GST formation in *A. annua*, despite several TFs having been confirmed to play important roles in regulating GST initiation, the underlying molecular mechanisms remain largely mysterious. As yet the core regulator, which plays a dominant role in GST formation, has not been identified. Single-cell RNA-sequencing (scRNA-seq) provides benefits to detect transcriptional heterogeneities in biological samples [179]. This would allow the discovery of new regulators related to GST formation, as well as artemisinin biosynthesis.

The advent of genome editing technologies has enabled us to precisely edit the *A. annua* genome. Therefore, understanding the molecular mechanism by which the expression of artemisinin biosynthetic genes declines during leaf age would provide targets for genome editing, leading to the generation of novel high-yielding *A. annua* germplasm. Zhou *et al.* have obtained gene knocking-out *A. annua* lines using the CRISPR/Cas9 system; however, the genome editing efficiency needs to be further improved [88]. In addition to knocking-out, *in-locus* editing such as targeted insertion of regulatory elements enables transcriptional and translational enhancement [180, 181]. We believe that the application of the recently developed plant biotechnologies would facilitate the germplasm innovation of high-artemisinin producing *A. annua* plants.

## Acknowledgements

This work was supported by the National Key Research and Development Program of China (2023YFC3503900), National Natural Science Foundation of China (82003889, 82304651), Zhejiang Provincial Natural Science Foundation of China (LQ21H280004), National ‘Ten-thousand Talents Program’ for Leading Talents of Science and Technology Innovation in China, National Young Qihuang Scholars Training Program, Innovative Leading Talents Program for Zhejiang Provincial Universities, the Major Science and Technology Projects of Breeding New Varieties of Agriculture in Zhejiang Province (2021C02074), Key Project at Central Government Level: The Ability Establishment of Sustainable Use for Valuable Chinese Medicine Resources (2060302), Research Project of Zhejiang Chinese Medical University (2021JKZDZC06, 2022RCZXZK23, 2023JKZKTS08) and China Postdoctoral Science Foundation (2022M722851). We appreciate the experimental support from the Public Platform of Pharmaceutical Research Center, Academy of Chinese Medical Sciences, Zhejiang Chinese Medical University.

## Author contributions

G.K., X.H,. and Y.L. conceived and designed the project. Y.L., X.H., and Y.Y. wrote the manuscript. X.H., L.L., K.T., and G.K. revised the manuscript. All authors read and approved the final manuscript.

## Data availability statement

Data availability does not apply to this review article as no new data were created or analysed in this study..

## Conflicts of interest

The authors have declared no conflict of interest.
